# May-Thurner syndrome: A case of extensive unprovoked left lower extremity deep vein thrombosis (DVT)

**DOI:** 10.1016/j.radcr.2023.11.027

**Published:** 2023-11-27

**Authors:** Akintayo Akinleye, Patrick Kwaah, Josephine Poku-Adusei, Hadiseh Kavandi, Katelyn Norman

**Affiliations:** a Department of Internal Medicine, Yale School of Medicine, Waterbury, CT, USA; b Department of Radiology, University of Maryland, Baltimore, MD, USA

**Keywords:** May-Thurner syndrome, Deep venous thrombosis, Anticoagulation, Thrombectomy, Iliac vein

## Abstract

May-Thurner syndrome (MTS) also known as iliac vein compression syndrome, is a congenital anatomical variant, that results from the extrinsic compression of the left common iliac vein by the right iliac artery with resultant formation of left venous thrombosis. We report a case of a young man with recurrent unprovoked left lower extremity DVT in the setting of May Thurner syndrome who required endovascular intervention and was discharged on oral anticoagulation.

## Background

May Thurner Syndrome (MTS) was first described by Rudolf Virchow in the early 1850s when he observed an increased incidence of the left iliofemoral vein compression by the right common iliac artery in a cadaveric study of patients with left iliofemoral thrombosis [1]. This condition was later described by May and Thurner in 1957 as autopsies on cadavers showed this same anatomic variant with intraluminal fibrous band in the left common iliac vein (CIV). The condition later became widely known by their names. The estimated prevalence of MTS in the general population is 14%-32%. It is however implicated in only 2%-5% of cases of lower limb deep vein thrombosis (DVT) [2,3]. Risk factors for MTS include female sex, multiparty, scoliosis, oral contraceptive use, cumulative radiation, and hypercoagulable disorders [4], [5], [6]. Development of this syndrome occurs in 3 stages; asymptomatic left CIV compression, formation of a venous spur, and finally left lower extremity DVT. Most patients with MTS never develop DVT [7]. Very few existing literatures have described recurrent DVT in the setting of May Thurner in young males.

We report a case of a young man with recurrent unprovoked left lower extremity DVT in the setting of May Thurner syndrome who required endovascular intervention and subsequently oral anticoagulation.

## Case presentation

A 20-year-old African American man with a past medical history of unprovoked left lower extremity DVT presented with acute psychosis and was incidentally found to have left lower extremity swelling and pain of a few days' duration. Medical history was significant for paranoid schizophrenia, poly-substance use disorder, obesity class II, and previous unprovoked left lower extremity DVT diagnosed 2 years prior.  He has poor medical follow-up and medication adherence. His medication regimen consisted of sodium valproate and benztropine.

On arrival at the hospital, his temperature was 36.7°C, heart rate of 113 beats per minute, respiratory rate of 19 cycles per minute, blood pressure 135/90 mm Hg, and oxygen saturation of 100% on room air.  Physical examination revealed a young obese man, alert and oriented, though reserved, with no respiratory distress.  The cardiopulmonary examination was unremarkable. The left leg, however, was swollen and tender, nonerythematous with palpable bilateral dorsalis pedis pulses.  Laboratory tests revealed normal complete blood count, initial hypokalemia of 3.1 mmol/L (range:3.5-5 mmol/L), bicarbonate of 22 mmol/L (range:22-28 mmol/L), and normal BUN and creatinine of 17 mg/dL (range:7-21 mg/dL) and 1.14 mg/dL (range:0.70-1.30 mg/dL) respectively.  Troponin I was mildly elevated at 0.072 ng/mL (range: ≤0.04 ng/mL) on 2 repeats. Electrocardiography [EKG] showed sinus tachycardia with no ST segment abnormalities.  An ultrasound of the left lower extremity uncovered a nonocclusive thrombus extending from the left external iliac to the popliteal vein.  Thrombus was also noted within the great and small saphenous veins.  CT of the abdomen and pelvis with IV contrast was requested to assess the proximal veins and revealed deep vein thrombosis involving the left common and superficial femoral veins, extending up into the left external iliac vein but without involvement of the left common iliac vein or IVC.  CT angiogram of the chest with IV contrast excluded pulmonary emboli.

Hypercoagulable work-up revealed elevated ANA of 1:2560 [Speckled pattern] and positive Rapid Plasma Reagin [RPR], though negative *Treponemal pallidum-*particle agglutination (TP-PA) assay, negative anti-CCP, antidouble-stranded DNA, anti-SSA, anti-SSB, anti-Smith, and rheumatoid factor.  Blood for inherited thrombophilia screen including factor V Leiden mutation, prothrombin gene [G20210A mutation], homocysteine level, protein C and S activity, Antithrombin III activity, lupus anticoagulant, antiphospholipid antibodies IgG, IgM and IgG and antibeta glycoprotein antibodies IgG, IgM and IgG revealed no abnormalities.

The patient was initially started on anticoagulation with IV heparin drip, which was transitioned to subcutaneous enoxaparin 1 mg/kg bd and eventually oral rivaroxaban to facilitate transfer to the inpatient psychiatric unit for stabilization.

**Fig. 1:**
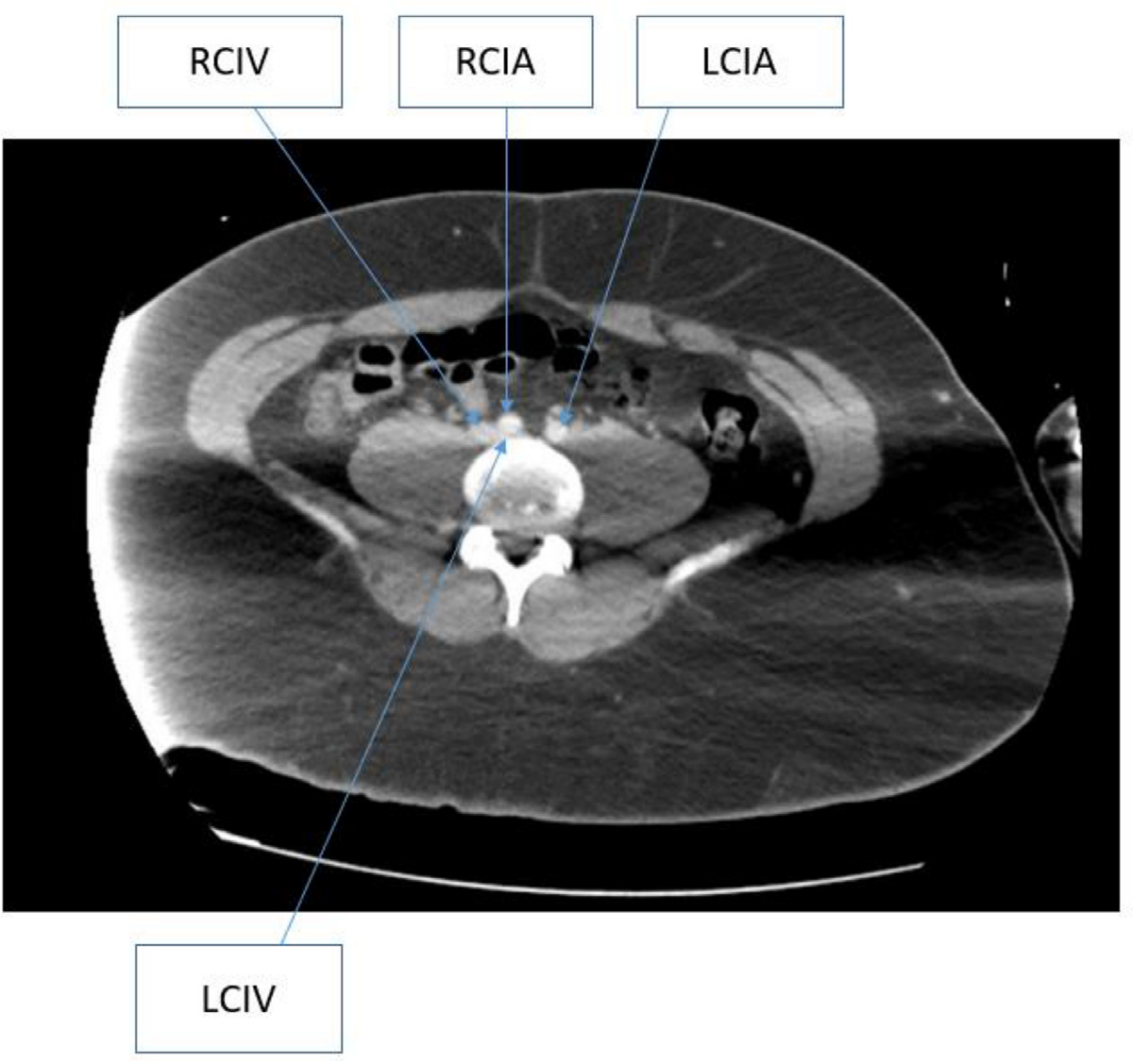
Contrast-enhanced CT of the abdomen and pelvis showed compression of the left common iliac vein by the right common iliac artery.

Following the transfer, however, he continued to experience worsening leg swelling and pain while also refusing oral anticoagulation.  The CT of the abdomen/pelvis was subsequently reviewed with the interventional radiologist with an eye toward possible thrombectomy given concerns regarding long-term anticoagulation adherence. This review of the left ileo-femoral DVT uncovered evidence of right common iliac artery compression on the left common iliac vein or May-Thurner Syndrome (Fig. 1). A repeat CT angiogram of the chest, ordered for persistent tachycardia, revealed no evidence of pulmonary thromboembolism to the level of the segmental pulmonary arteries. The main pulmonary artery was noted to be dilated suspicious for pulmonary arterial hypertension raising concern for chronic thromboembolism.

TTE (Transthoracic echocardiography) showed normal global left ventricular systolic function with an ejection fraction of 55%-60%, normal left ventricular diastolic function with normal left atrial pressure.  Furthermore, there was no valvular abnormality, normal pulmonary artery systolic pressure (23 mm Hg) and there was dilatation of the ascending aorta.

**Fig. 2:**
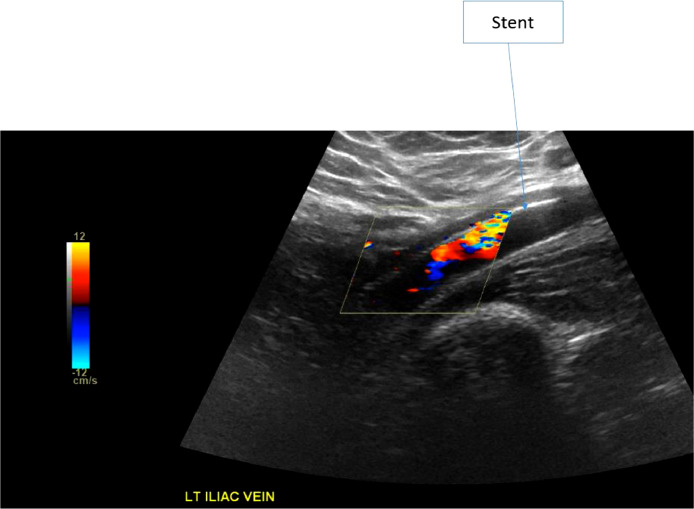
Venous Doppler Ultrasound of the left iliac vein with self-expanding stents.

The patient underwent ultrasound-guided left popliteal vein access venogram with mechanical venous thrombectomy of the left common iliac vein, left external iliac vein, and left common femoral vein, as well as balloon angioplasty of the left common iliac vein, left external iliac vein, left common femoral vein, left femoral and left popliteal veins and placement of self-expanding stents in the left common iliac vein, left external iliac vein and left common femoral vein (Fig. 2). There was no insertion of a prophylactic IVC filter. Anticoagulation was initially IV unfractionated heparin drip and was transitioned to oral anticoagulation and aspirin.

The patient was discharged back to the inpatient psychiatric unit after a few days with outpatient hematology and vascular surgery follow-up.

## Discussion

In young patients presenting with DVT, a detailed history, physical examination, and diagnostic workup are warranted. Thrombophilia workup should always be done to identify the underlying cause of the DVT [3]. The presence of known risk factors or positive results from thrombophilia workup may halt further investigation for this anatomic variant [8]. Risk factors of MTS share with DVT include hypercoagulable disorder, oral contraceptive use, and dehydration and this may explain why it is overlooked when DVT is identified in these settings [8]. Our patient is a young male with none of these risk factors.

Prior studies by Bhadra et al. in 2019 described a 21-year-old male with no risk factors presenting with left lower extremity DVT complicated by acute bilateral pulmonary embolism in the setting of MTS. They recommended considering and investigating MTS in young males with recurrent or unprovoked left sided DVT as it may require long-term anticoagulation and possibly endovascular intervention [8]. Author Hng in 2021 also described a 23-year-old male with no identifiable risk factors who presented with MTS and DVT. The patient was managed initially with anticoagulation therapy and later transferred for endovascular intervention. They, therefore, advocated that patients presenting with DVT of the left lower limb should be investigated with duplex ultrasound and/or CT venography for MTS particularly if no other cause has been identified [9]. Our study seeks to also affirm these 2 earlier recommendations and shed more light on the need to consider May Thurner in young males especially those without any known risk factors. The diagnosis of MTS is made by imaging. Imaging modalities used to diagnose MTS include Doppler USG, plethysmography, CT venography, MR venography, and conventional venography. Contrast venography is considered the gold standard modality for MTS [7]. In our study, however, the imaging modalities used were USG and CT of the abdomen and pelvis with contrast. USG is often the first-line technique used in diagnosing DVT. Although it is the most common imaging modality, it has several limitations in diagnosing MTS. These limitations include technical difficulties in assessing the iliac veins and inferior vena cava and false positive high flow velocity in the common iliac vein which may indicate obstruction or compression [1,10]. In our case, USG demonstrated DVT but failed to identify the compression of the iliac veins.

CT venography has a high sensitivity and specificity to detect MTS. It has other advantages including its ability to rule out extrinsic compressions like lymphadenopathy and hematoma, identify acute DVT, and outline collateral pathways. CT also eliminates the need for technical expertise. It has limitations in pregnancy due to radiation dose and can overestimate the degree of compression in dehydrated patients [2,^11^,12]. With our patient, CT with contrast accurately diagnosed MTS hence the use of this imaging modality in this condition is justified.

May Thurner anatomy only requires intervention when symptomatic. The mainstay of management of MTS is the removal of the clot with pharmaco-chemical thrombolysis and mechanical thrombectomy to prevent post-thrombotic syndrome [13,14]. Catheter-directed thrombolysis with anticoagulation in iliofemoral DVT is superior to anticoagulation alone. Anticoagulation on its own is insufficient to manage MTS with DVT [15,16]. Prophylactic IVC filter placement prior to clot removal is not recommended [17]. Unfractionated heparin has an increased risk of bleeding due to heparin-induced thrombocytopenia, so it is less preferred to LMWH and fondaparinux. Factor Xa inhibitors, particularly rivaroxaban, have been shown to be safe in the management of iliofemoral vein thrombosis [18,19].

## Patient consent

An informed consent was obtained from the patient for this case report prior to submission in your journal.
